# IRS1/PI3K/AKT pathway signal involved in the regulation of glycolipid metabolic abnormalities by Mulberry (*Morus alba* L.) leaf extracts in 3T3-L1 adipocytes

**DOI:** 10.1186/s13020-019-0281-6

**Published:** 2020-01-02

**Authors:** Qinghai Meng, Xu Qi, Ying Chao, Qi Chen, Peng Cheng, Xichao Yu, Meiyu Kuai, Jingzhen Wu, Wenwen Li, Qichun Zhang, Yu Li, Huimin Bian

**Affiliations:** 1 School of Pharmacy, Nanjing University of Chinese Medicine, Nanjing, 210023 China; 2 School of Medicine and Life Sciences, Nanjing University of Chinese Medicine, Nanjing, 210023 China; 3 Jiangsu Key Laboratory for Pharmacology and Safety Evaluation of Chinese Materia Medica, School of Pharmacy, Nanjing University of Chinese Medicine, Nanjing, 210023 China; 4 Department of Respiratory Medicine, The First Affiliated Hospital of Nanjing Medical University, Nanjing, 210029 China

**Keywords:** Mulberry leaf, Flavonoid, Adipocytes, Type 2 diabetes, PI3K/AKT

## Abstract

**Background:**

Mulberry (*Morus alba* L.) leaf tea benefits the control of diabetes in Asian nations. This study was aim to investigate if the flavonoids, which extracts from mulberry leaves, could regulate the metabolism of glycolipid, and to investigate if flavonoids could regulate IRS1/PI3K/AKT pathway signal to affect the expression of FAS and membrane transfer capacity GLUT4 in 3T3-L1 adipocytes.

**Results:**

Results revealed that flavonoids decreased the levels of free fatty acid and increased the glucose consumption and the levels of adiponectin and leptin in a dose-dependent manner, and remarkably increased the protein expression levels of p-IRS1, p-PI3K, p-Akt, total GLUT4, and membrane GLUT4, and decreased the protein expression levels of PTEN and FAS in 3T3-L1 adipocytes IR model. On the other hand, wortmannin (2 nM), a selective and irreversible PI3K inhibitor, significantly decreased the glucose consumption and the adiponectin and leptin levels, and increased the free fatty acid level in flavonoids treated 3T3-L1 adipocytes IR model. Furthermore, wortmannin (2 nM) partly eliminated the activation of PI3K/AKT signaling, the suppression of FAS, and the up-regulated membrane transfer capacity of GLUT4 in flavonoids treated 3T3-L1 adipocytes IR model.

**Conclusion:**

In conclusion, our results illustrated that mulberry leaf extracts flavonoids alleviated the glycolipid metabolic abnormalities in 3T3-L1 adipocytes IR model, and the effect was associated with the activation of IRS1/PI3K/AKT pathway, the suppression of FAS, and the up-regulation of membrane transfer capacity of GLUT4.
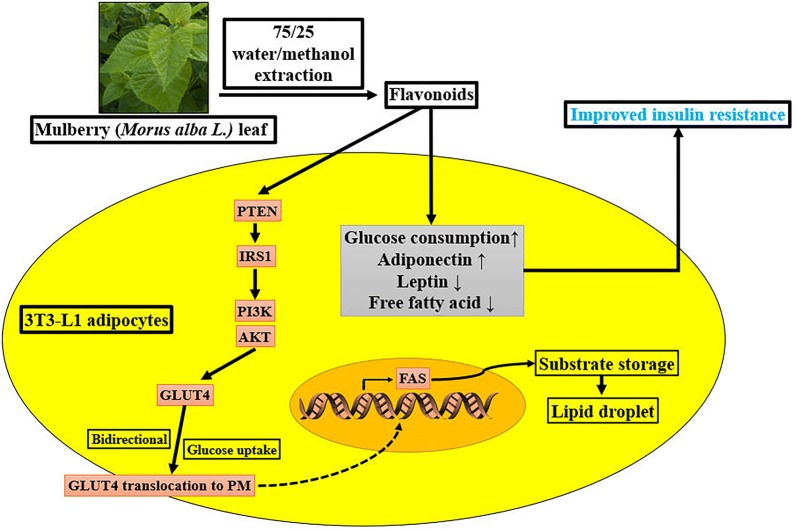

## Introduction

As one of the main factors in the pathogenesis of type 2 diabetes mellitus (T2DM), insulin resistance (IR) is characterized by the decline of insulin sensitivity in insulin target organs [1]. Adipose tissue, which is mainly made up of adipocytes, plays an important role in IR and T2DM [2]. Moreover, as the largest energy storage organ of human body, the endocrine ability of adipocytes to secrete adipocytokines (such as adiponectin, leptin) were aberrant when comes to IR [3]. Current study testified that disruption of the balance of insulin signaling pathway is one of the main cause of IR in adipose tissue [4] ,such as the IRS1/PI3K/AKT signaling pathway [5]. As a downstream factor of IRS1/PI3K/AKT signaling pathway, the impaired translocation ability of GLUT4 is also a cause of IR and T2DM [6]. Therefore, the activation of insulin signaling molecules in adipose tissue and fat cells to promote the translocation of intracellular GLUT4 to the cell membrane and to increase the uptake and consumption of glucose may be a new strategy for the treatment of T2DM [7]. In our previous studies, we testified that the flavonoids that came from mulberry leaves could reduce blood glucose and adipocytes hypertrophy in mice [8].

Study reports that mulberry leaf extract stimulates glucose uptake and GLUT4 translocation in rat adipocytes [9], however, the mechanism of the hypoglycemic effects of the flavonoids that came from mulberry leaves in adipocytes was still unclear. Here, we used a 3T3-L1 adipocytes IR model to evaluate the effects of the flavonoids on the metabolism of glycolipid, and to investigate if the flavonoids could regulate IRS1/PI3K/AKT pathway signal to affect the expression of FAS and the membrane transfer capacity of GLUT4.

## Methods

### Reagents

Dulbecco’s modified Eagle’s medium (DMEM), fetal bovine serum (FBS), bovine calf serum (BCS), penicillin and streptomycin mixture were obtained from Gibco (Grand Island, NY, USA). 3-[4,5-dimethylthiazol-2-yl]-2,5-diphenyltetrazolium bromide (MTT), dimethylsulfoxide (DMSO), 3-isobutyl-1-methylxanthine (IBMX), dexamethasone (DEX), and insulin were purchased from Sigma Aldrich (St Louis, MO, USA). Free fatty acid, adiponectin, and leptin Enzyme-linked immunosorbent assay (ELISA) kits were purchased from YiFeiXue Biotechnology (Nanjing, China) and Jian-Cheng Biotechnology (Nanjing, China). GLUT4 rabbit antibody, anti-PTEN antibody, anti-Fatty Acid Synthase antibody and Wortmannin were purchased from Abcam Biotechnology (Cambridge, UK). Total Akt rabbit polyclonal antibody and p-Akt-ser473 rabbit polyclonal antibody were supplied by Santa Cruze (California, USA). Goat anti-rabbit IgG (H + L) dylight 488 was supplied by Bioworld (Nanjing, China). Total PI3K rabbit polyclonal antibody, p-PI3K rabbit polyclonal antibody, total IRS1 rabbit polyclonal antibody, p-IRS1-ser307 rabbit polyclonal antibody, Na–K-ATPase rabbit antibody, β-actin rabbit polyclonal Antibody, and goat anti-rabbit IgG antibody were all provided by Nanjing Enjing Biotechnology (Nanjing, China).

### Preparation and quality control of mulberry leaf extracts flavonoids

**Fig. 1:**
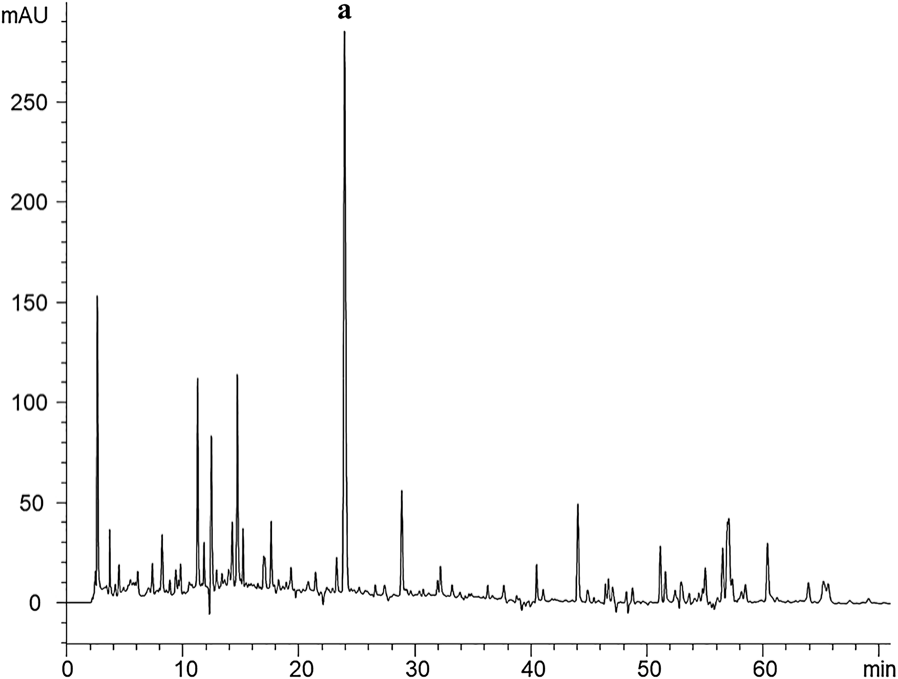
Representative HPLC chromatograms of flavonoids that extracts from mulberry leaves. a: Rutin

The mulberry leaves were provided by the Beijing Tong Ren Tang Co. Ltd (Beijing, China). The flavonoids extract of mulberry leaves was prepared with the following procedure that we have reported before [10]. The main ingredient of rutin in mulberry leaf extracts flavonoids (1 g/mL) were determined using an Agilent 1260 liquid chromatography system. The procedure of HPLC chromatograms analysis of flavonoids that extracts from mulberry leaves could been seen in our previous studied [10]. Here we provided a representative HPLC chromatograms of flavonoids that extracts from mulberry leaves, it can be seen in Fig. 1.

### Cell culture and the differentiation of 3T3-L1 preadipocytes

3T3-L1 preadipocytes were obtained from American Type Culture Collection (ATCC, Manassas, VA, USA) (2 to 5 generations). The cells were grown in DMEM supplemented with 10% FBS, 100 U/mL of penicillin and 100 μg/mL of streptomycin under a humidified atmosphere with 5% CO_2_ at 37 °C.

The 3T3-L1 preadipocytes were incubated in differentiation medium containing 0.5 μM IBMX, 100 nM insulin and 1 μM dexamethasone (DEX) in DMEM containing 10% FBS to induce differentiation. After 2–3 days, the differentiation medium was changed to DMEM medium containing 100 nM insulin and 10% FBS (maintenance medium). The medium was replaced with fresh maintenance medium for every 2 days. Adipocytes were used for experiments on the 8–9th day form the initial day of differentiation.

### The establishment of the IR model of 3T3-L1 adipocytes and reagents treatment

After the adipocytes were mature, different concentrations (0.01, 0.1, 1, 10 μM) of DEX was added into different groups. Adipocytes without DEX set as control group. Then they were cultured at 5% CO_2_ and 37 °C for 24 h. The content of glucose in the supernatant was measured according to the manufacture’s manual. The formula was as follow: Glucose consumption = (25 − OD value of measurement pore)/OD value of standard pore × 5.55 mol/L; Decrement of glucose consumption = Glucose consumption of one group − Glucose consumption of control group. The most suitable concentration of DEX was selected based on the decrement of glucose consumption in different groups.

After confirming the concentration of DEX. A concentration (1 μM) of DEX was added into3T3-L1 adipocytes. Then they were cultured at 5% CO_2_ and 37 °C. The glucose consumption was calculated at different times (3 h, 6 h, 12 h, 24 h, 36 h, and 48 h). Decrement of glucose consumption was calculated. A proper duration of DEX (1 μM) incubation was selected based on the decrement of glucose consumption in different time compared to the control group without DEX stimulation. The cells were co-treated with flavonoids at different concentrations or other reagents in the 24 h. In other in vitro experiments, the concentration of flavonoids was 20 μg/ml and the concentration of wortmannin was 2 nM.

### MTT assay

3T3-L1 preadipocytes were added into 96-well plate (1 × 10^4^ cells/mL) and cultured with serum-free culture medium overnight. After incubation, the cells were treated with or without flavonoids at different concentrations for 24 h at 37 °C in 5% CO_2_. Then MTT (5 mg/mL) was added to each well, and the plate was incubated at 37 °C in the dark for 4 h. The supernatant of each well was discarded, and 150 μL of DMSO was added to each well. The absorbance value was measured by the microplate reader a Spectra Max M2 microplate reader (Molecular Devices, Sunnyvale, CA, USA) at 490 nm.

### Supernatant ELISA detection

Samples of 0.5 ml of the supernatant of the cell-cultured medium were obtained from the different groups. The supernatant was separated and collected by centrifugation. The samples were then assessed for glucose content, free fatty acid, adiponectin and leptin using an ELISA kit, according to the manufacturer’s instructions.

### Oil Red O staining

3T3-L1 cells were washed with phosphate-buffered saline (PBS) and fixed with 10% formalin-PBS solution for 10 min. After removing this solution, the differentiated cells were stained with Oil Red O dye (Sigma Aldrich, St. Louis, MO, USA) for 10 min at room temperature. The cells were washed four times with distilled water, and the images were collected under a microscope.

### Western bolt assay

Before the adipocytes from different groups were lysed in RIPA buffer, the cells were stimulated by 100 nM insulin for 10 min. After that, the samples were then centrifuged (4 °C, 12,000 rpm, 15 min) and the supernatants were collected. The separation of membrane proteins from the cells was performed refer to the directions prepared by the manufacturer of the membrane protein extraction kit (Beyotime Biotechnology, Shanghai, China). The concentration of the protein in the supernatant was determined using BCA protein assay reagent (Beyotime Biotechnology, Shanghai, China). Equal amounts of protein (30–50 μg) in different groups were electrophoresed in SDS-PAGE gel, and then transferred to PVDF membranes (Millipore, Billerica, MA, USA). The PVDF membranes were blocked by 5% non-fat milk powder in TBS-T buffer for 2 h at room temperature, and then incubated with various primary antibodies for overnight at 4 °C. After washing with TBS-T buffer, the PVDF membranes were incubated with appropriate secondary antibodies (dilute 1:10,000) for 2 h at room temperature. Protein bands were detected by enhanced chemiluminescence method using the ECL kit. The protein bolts were visualized by autoradiography, and the bolts was quantified via a Gel Pro analyzer. Normalization of protein expression was carried out using β-actin.

### Immunofluorescence staining

Adipocytes were growing on glass coverslips, treated with different reagents according to the groups, and followed by 100 nM insulin treatment for 10 min. Glass coverslips with cells were fixed by 4% paraformaldehyde and washed 3 times with PBS. Subsequently, they were incubated with p-IRS1, p-PI3K, p-Akt and GLUT4 antibodies followed by fluorescein-conjugated secondary antibodies incubated in the blocking solution. Then the nuclei were stained with DAPI for 5 min. After 3 times washing with PBS, photos were taken with the ZEN 2011 imaging software on a Zeiss invert microscope (CarlZeiss, Hallbergnoos, Germany) under 100-fold magnification.

### Statistical analysis

SPSS 21.0 (SPSS, Inc., Chicago, IL, USA) was used for all the statistical analysis. Data were expressed as mean ± standard deviation (SD). Differences were evaluated using either two-tailed Student’s *t* test or one-way ANOVA followed by post hoc Dunnett’s test. P < 0.05 was different with statistical significance between groups.

## Results

### The establishment of the IR model of 3T3-L1 adipocytes

**Fig. 2:**
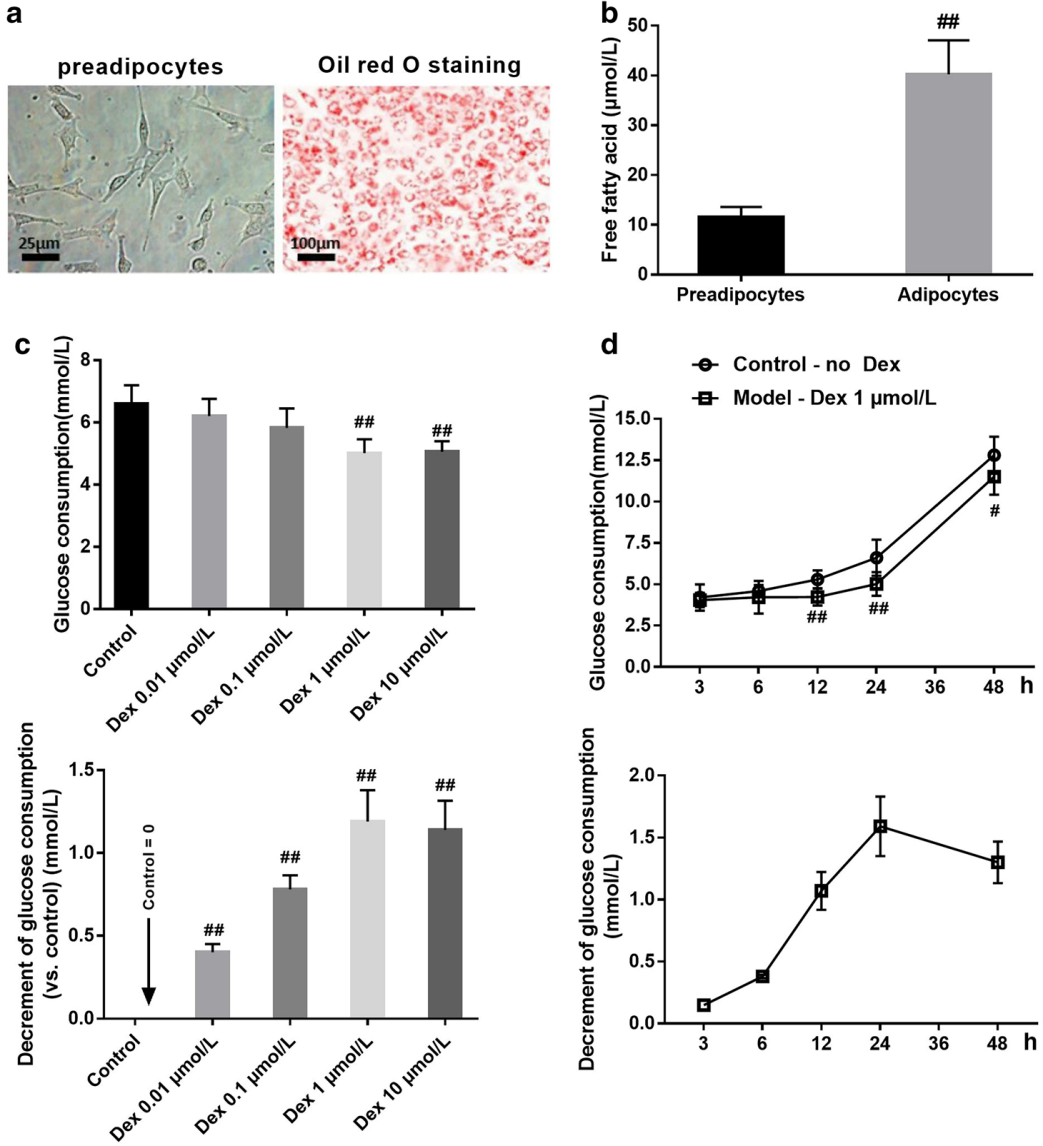
The establishment of the IR model of 3T3-L1 adipocytes. **a** The inducing of 3T3-L1 preadipocytes for 8 days and the identification of mature adipocytes by oil red O staining (×200). **b** The free fatty acid of 3T3-L1 preadipocytes and adipocytes. (n = 10). Comparison in two groups, ^##^p < 0.01. **c** The effects of different concentrations of dexamethasone on glucose consumption (up panel) and consumption decrement (down panel) in 3T3-L1 adipocytes (n = 10). ^##^p < 0.01, compared with control group. **d** The effects of 1 μmol/L dexamethasone on glucose consumption and glucose consumption decrement for different time in 3T3-L1 adipocytes (n = 10). Comparison in two groups, ^##^p < 0.01

The morphology of 3T3-L1 preadipocytes showed that the cells were typical spindle type, and there were no fat drops in the cytoplasm (Fig. 2a). On the eighth day after induction of differentiation, and the cells were stained with oil red O dye, showing that the 3T3-L1 preadipocytes had been differentiated into mature fat cells (Fig. 2a).

Free Fatty acid content assay showed that 3T3-L1 adipocytes had more free fatty acids than 3T3-L1 preadipocytes (Fig. 2b). After treatment with different concentrations of DEX for 24 h in mature adipocytes, the glucose content in the cell-cultured medium supernatant was determined by glucose oxidase–peroxidase method and the glucose consumption and decrement of glucose consumption were calculated. The results showed that the DEX existing could decrease the glucose consumption (Fig. 2c, up panel) and the decrement of glucose consumption. 1 μmol/l concentration of DEX showed a maximum decrement of glucose consumption (Fig. 2c, down panel).

Next, we observed the effect of 1umol/l DEX on glucose consumption at different time. The results showed that DEX could reduce glucose consumption within 48 h. The IR circumstance sustained for at least 48 h (Fig. 2d, up panel). The decrement of glucose consumption arrived to a platform period at 24 h (Fig. 2d, down panel).

### The abatement of metabolic abnormalities caused by the flavonoids in IR model of 3T3-L1 adipocytes

**Fig. 3:**
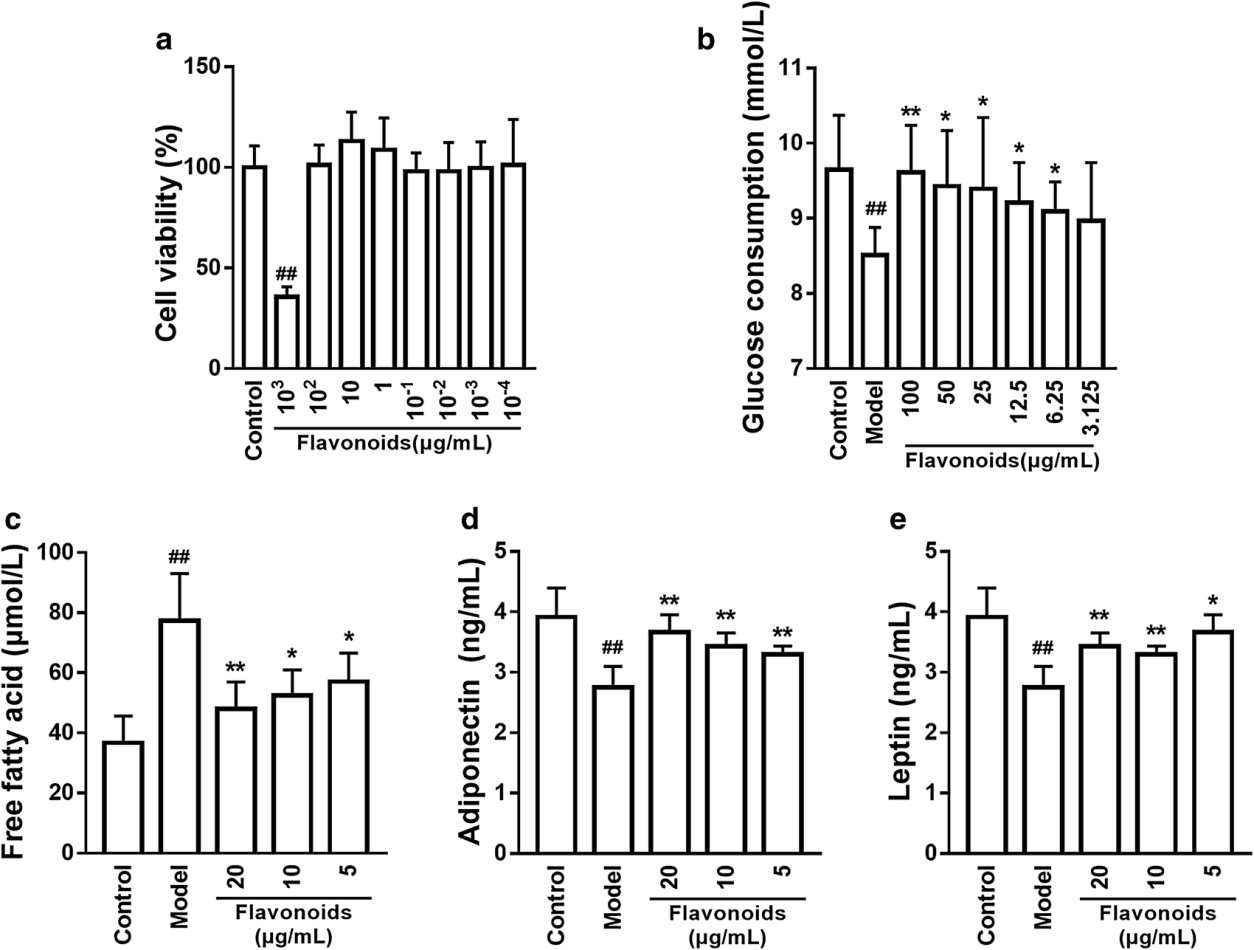
The flavonoids regulate glucose and lipid metabolism in IR model of 3T3-L1 adipocytes. **a** Different concentrations of flavonoids on the proliferation of 3T3-L1 preadipocytes. **b** Different concentrations of flavonoids on the glucose consumption of 3T3-L1 dipocytes. **c** Different concentrations of flavonoids on the free fatty acid of 3T3-L1 dipocytes. **d** Different concentrations of flavonoids on the adiponectin of 3T3-L1 dipocytes. **e** Different concentrations of flavonoids on the leptin of 3T3-L1 dipocytes. n = 10. ^##^p < 0.01, compared with control group; *p < 0.05, **p < 0.01, compared with model group

In the concentration range of 1000–0.0001 μg/ml of flavonoids had no influence on 3T3-L1 cell viability (p > 0.05) (Fig. 3a). Furthermore, the concentration range of 100–6.25 μg/ml of flavonoids significantly increased glucose consumption in IR model of 3T3-L1 adipocytes (p < 0.05, p < 0.01) (Fig. 3b). Thus, we used the concentrations of 20, 10, and 5 μg/ml of flavonoids to do the subsequent experiments. The results revealed that the flavonoids decreased the levels of free fatty acid (Fig. 3c) and increased the levels of adiponectin (Fig. 3d) and leptin (Fig. 3e) in IR model of 3T3-L1 adipocytes (p < 0.05, p < 0.01).

### The flavonoids activate IRS1/PI3K/AKT signaling in IR model of 3T3-L1 adipocytes

**Fig. 4:**
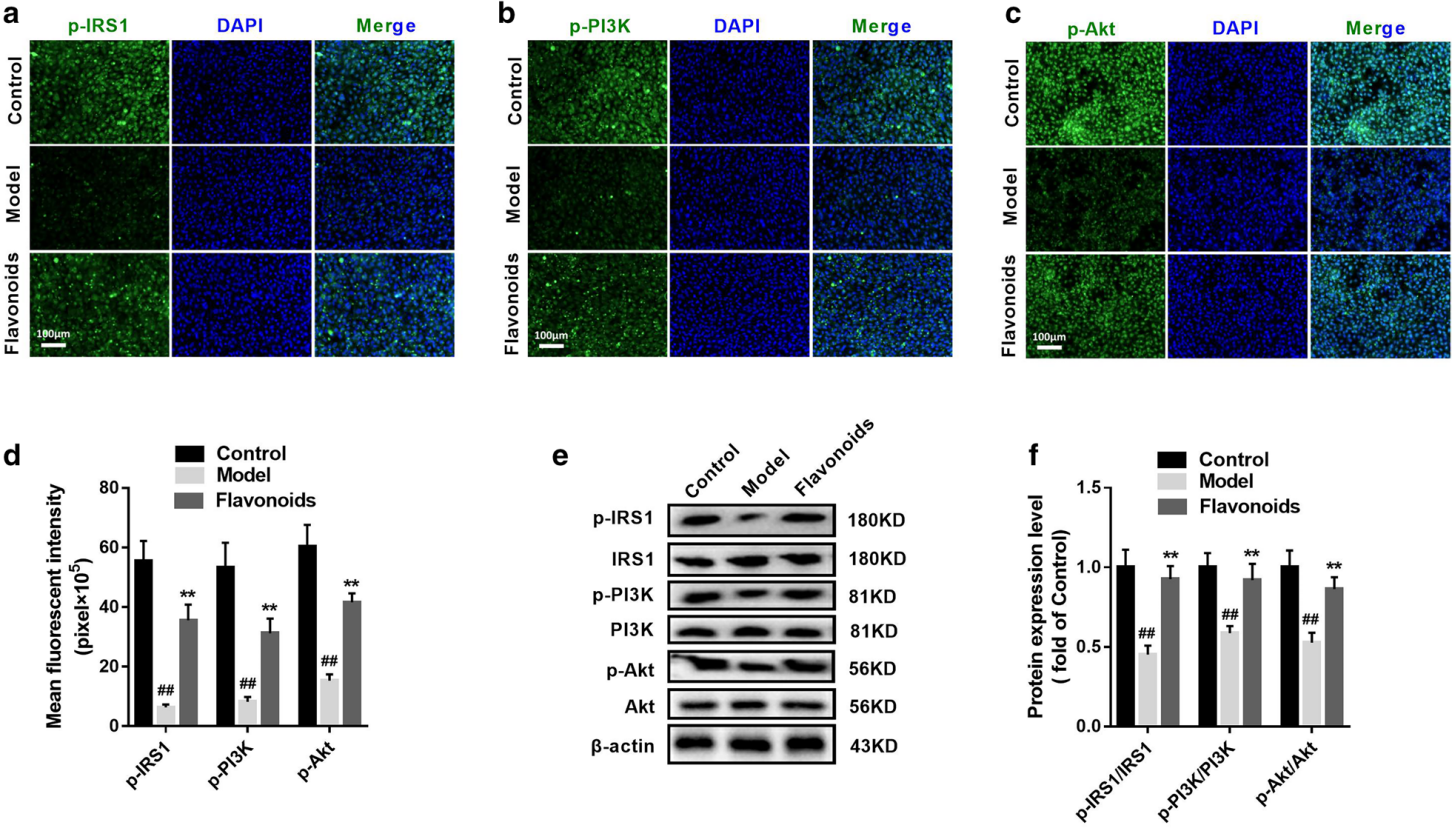
The flavonoids activate IRS1/PI3K/AKT signaling in IR model of 3T3-L1 adipocytes. **a** Immunofluorescent staining of p-IRS1 in3T3-L1 dipocytes (100 ×). **b** Immunofluorescent staining of p-PI3K in 3T3-L1 dipocytes (×100). **c** Immunofluorescent staining of p-Akt in 3T3-L1 dipocytes (×100). **d** Related to **a**–**c**, the quantitative of immunofluorescent staining of p-IRS1, p-PI3K and p-Akt in 3T3-L1 dipocytes. n = 6. ^##^p < 0.01, compared with control group; **p < 0.01, compared with model group. **e** The representative result of western blot analysis of p-IRS1, IRS1, p-PI3K, PI3K, p-Akt and Akt in 3T3-L1 dipocytes. **f** Related to e, the quantitative of p-IRS1, IRS1, p-PI3K, PI3K, p-Akt and Akt protein expressions in 3T3-L1 dipocytes. n = 6. ^##^p < 0.01, compared with control group; **p < 0.01, compared with model group

Immunofluorescence and western blotting revealed that the protein expression levels of p-IRS1, p-PI3K, and p-Akt in the model group were significantly lower than those in the control group in 3T3-L1 adipocytes (p < 0.01) (Fig. 4). After administration of flavonoids (20 μg/ml), the protein expression levels of p-IRS1, p-PI3K, and p-Akt were significantly increased compared to the model group (p < 0.01) (Fig. 4). Therefore, the IRS1/PI3K/AKT signaling was significantly aroused by flavonoids in IR model of 3T3-L1 adipocytes.

### The flavonoids elevate GLUT4 capacity in IR model of 3T3-L1 adipocytes

**Fig. 5:**
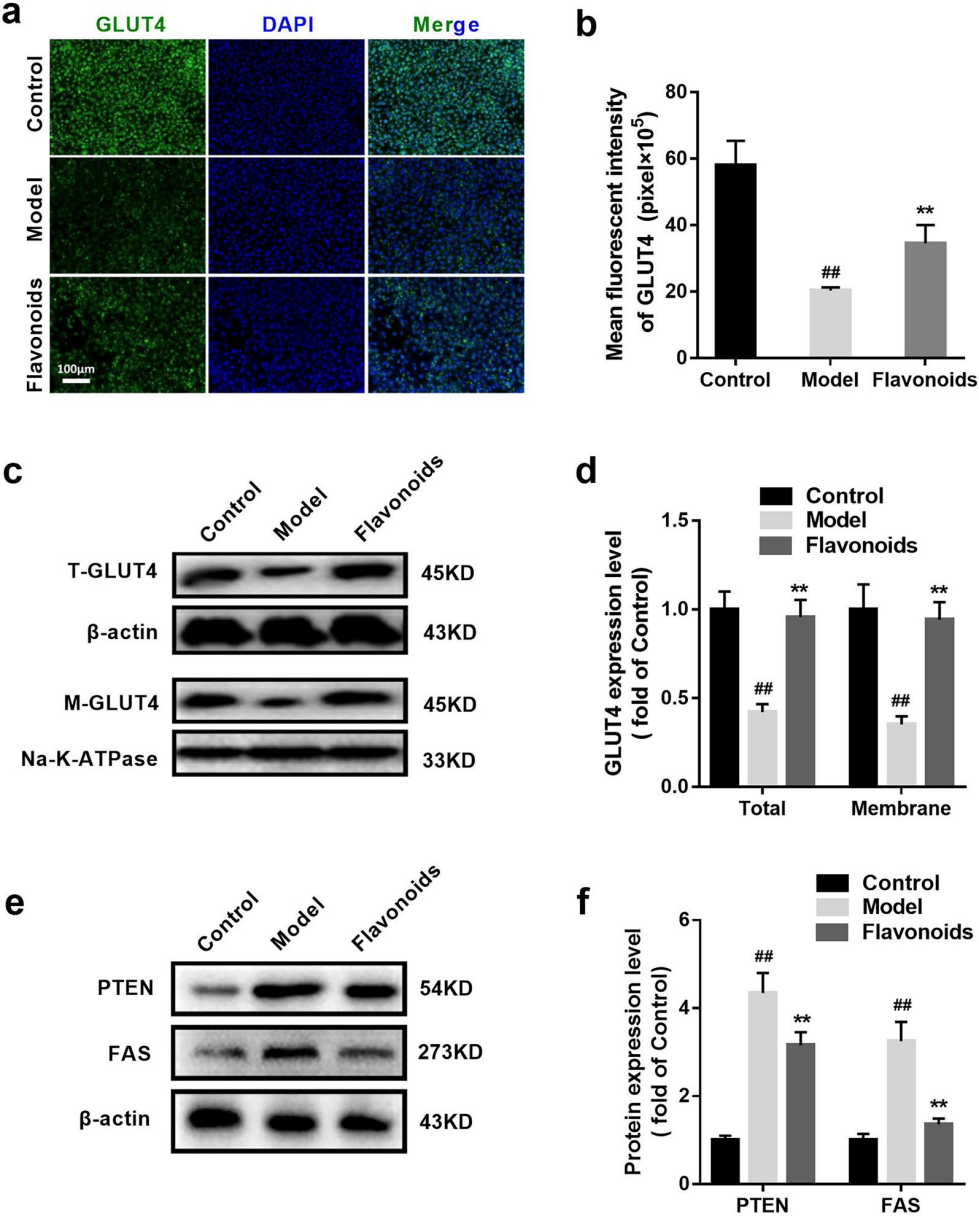
The flavonoids elevate GLUT4 expression in IR model of 3T3-L1 adipocytes**. a** Immunofluorescent staining of GLUT4 in 3T3-L1 dipocytes (×100). **b** Related to a, the quantitative of immunofluorescent staining of GLUT4 in 3T3-L1 dipocytes. n = 6. ^##^p < 0.01, compared with control group; **p < 0.01, compared with model group. **c** The representative result of western blot analysis of the total GLUT4 and the membranes of GLUT4 in 3T3-L1 dipocytes. **d** Related to c, the quantitative of the total GLUT4 and the membranes of GLUT4 protein expressions in 3T3-L1 dipocytes. n = 6. ^##^p < 0.01, compared with control group; **p < 0.01, compared with model group. **e** The representative result of western blot analysis of the PTEN and FAS in 3T3-L1 dipocytes. **f** Related to e, the quantitative of the PTEN and FAS protein expressions in 3T3-L1 adipocytes. n = 6. ^##^p < 0.01, compared with control group; **p < 0.01, compared with model group

To test the effects of flavonoids on GLUT4 vitality, we measured the expressions of GLUT4 in cytomembrane and total cell in 3T3-L1 adipocytes. The results of immunofluorescence and western blot showed that the expression levels of total GLUT4 in the model group were lower than those in the control group (p < 0.01) (Fig. 5). The flavonoids (20 μg/ml) reduced the expression level of total GLUT4 in IR model of 3T3-L1 adipocytes (Fig. 5). Western blot showed that the same change happened to the expression level of membrane GLUT4 (Fig. 5c, d). Thus, suggesting that flavonoids elevate GLUT4 capacity in IR model of 3T3-L1 adipocytes. Western blot also showed that the PTEN and FAS expressions in the model group were higher than that in the control group (p < 0.01), and flavonoids (20 μg/ml) reduced the PTEN and FAS expressions in IR model of 3T3-L1 adipocytes (Fig. 5e, f). Thus, the negative modulator antagonizing the IRS1/PI3K/AKT signaling, PTEN and the synthesis of fatty acids were suppressed by flavonoids in IR model of 3T3-L1 adipocytes.

### The inhibition of PI3K partly eliminates the activation of flavonoids on PI3K/AKT signaling and GLUT4 capacity in IR model of 3T3-L1 adipocytes

**Fig. 6:**
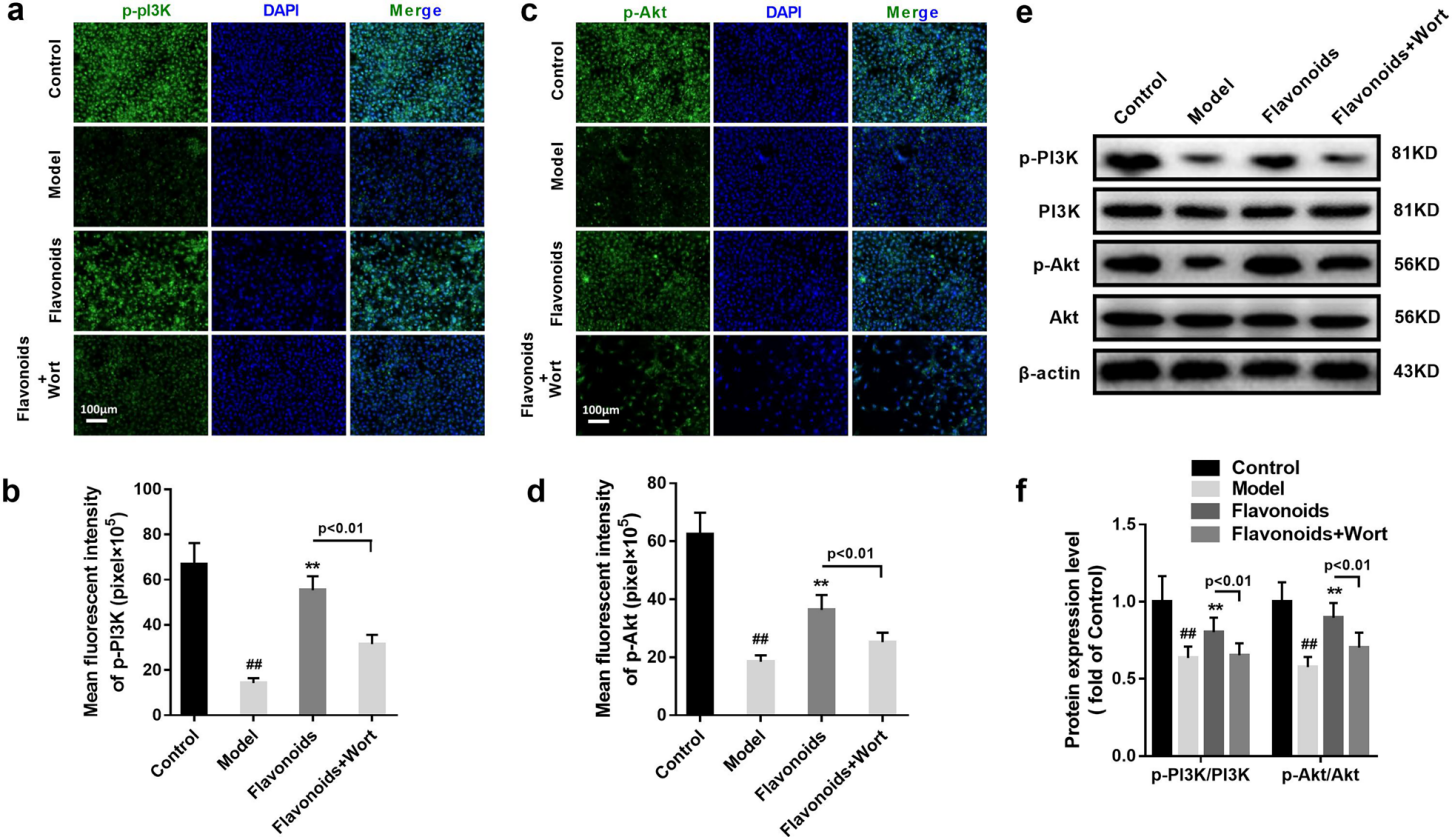
The inhibition of PI3K partly eliminated the activation of flavonoidson PI3K/AKT signaling in IR model of 3T3-L1 adipocytes. **a** Immunofluorescent staining of p-PI3K in 3T3-L1 dipocytes (×100). **b** Related to a, the quantitative of immunofluorescent staining of p-PI3K in 3T3-L1 dipocytes. **c** Immunofluorescent staining of p-AKT in 3T3-L1 dipocytes (×100). **d** Related to c, the quantitative of immunofluorescent staining of p-AKT in 3T3-L1 dipocytes. **e** The representative result of western blot analysis of the total p-PI3Kandp-AKT in 3T3-L1 dipocytes. **f** Related to e, the quantitative of the p-PI3K and p-AKT protein expressions in 3T3-L1 dipocytes. n = 6. ^##^p < 0.01, compared with control group; **p < 0.01, compared with model group

**Fig. 7:**
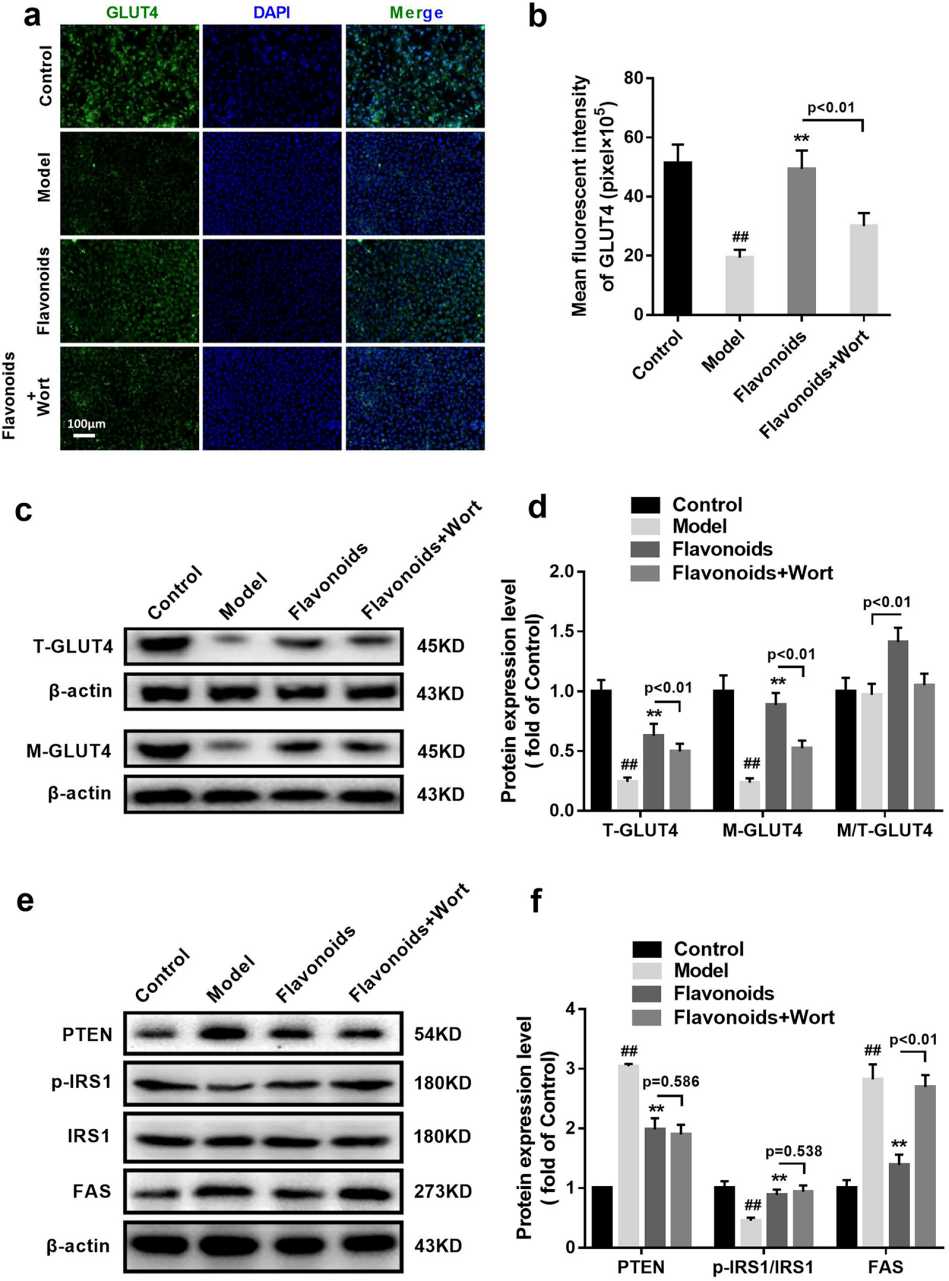
The inhibition of PI3K partly eliminated the activation of flavonoids on GLUT4 capacity in IR model of 3T3-L1 adipocytes. **a** Immunofluorescent staining of GTUT4 in 3T3-L1 dipocytes (×100). **b** Related to a, the quantitative of immunofluorescent staining of GLUT4 in 3T3-L1 dipocytes. **c** The representative result of western blot analysis of the total GLUT4 and the membranes of GLUT4 in 3T3-L1 dipocytes. **d** Related to c, the quantitative of the total GLUT4 and membranes of GLUT4 protein expressions in 3T3-L1 dipocytes. **e** The representative result of western blot analysis of the PTEN, p-IRS1, IRS1, and FAS in 3T3-L1 dipocytes. **f** Related to e, the quantitative of PTEN, p-IRS1/IRS1, and FAS protein expressions in 3T3-L1 dipocytes. n = 6. ^##^p < 0.01, compared with control group; **p < 0.01, compared with model group

To further assess the effects of flavonoids on PI3K/AKT signaling and GLUT4 capacity in IR model of 3T3-L1 adipocytes, wortmannin (2 nM), a selective and irreversible PI3K inhibitor, was used to inhibit the activation of PI3K. Immunofluorescence and western blot showed that, in flavonoids treated IR model of 3T3-L1 adipocytes, wortmannin remarkably reduced the expression levels of p-PI3K, p-Akt, total GLUT4 and membrane GLUT4 in IR model of 3T3-L1 adipocytes (p < 0.01) (Figs. 6d, 7a). Interestingly, Western blot showed that wortmannin remarkably increased the FAS expression (p < 0.01), but had little influence on PTEN and p-IRS1 expressions in IR model of 3T3-L1 adipocytes when flavonoids treatment existing (Fig. 7e, f). These results might indicate that the inhibition of PI3K decreased the activation of flavonoids on PI3K/AKT signaling and GLUT4 capacity in IR model of 3T3-L1 adipocytes.

### The inhibition of PI3K alleviates the impacts of flavonoids on the abatement of metabolic abnormalities in IR model of 3T3-L1 adipocytes

**Fig. 8:**
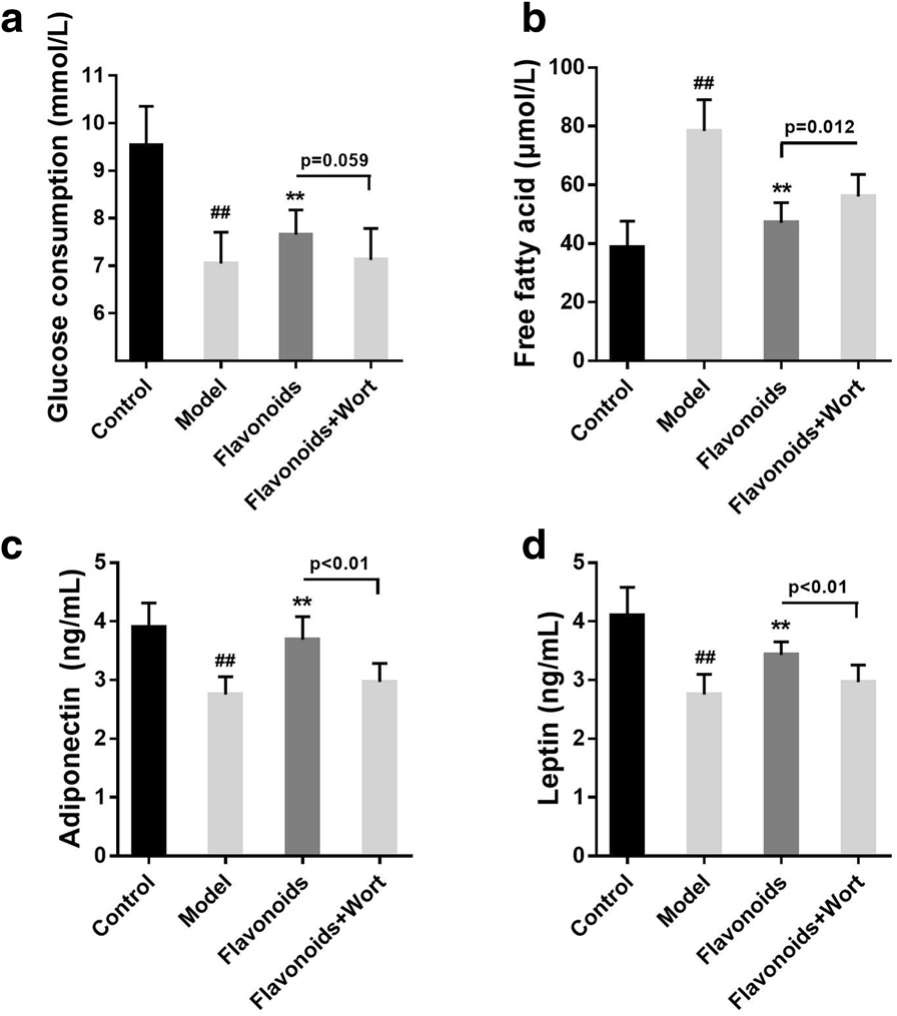
The inhibition of PI3K alleviates the impacts of flavonoids on the abatement of metabolic abnormalities in IR model of 3T3-L1 adipocytes. **a** Thetreatment of flavonoidson the glucose consumption of 3T3-L1 dipocyteswith or without wortmannin. **b** Thetreatmentof flavonoidson the free fatty acid of 3T3-L1 dipocyteswith or without wortmannin. **c** Thetreatment of flavonoidson the adiponectin of 3T3-L1 dipocyteswith or without wortmannin. **d** The treatmentof flavonoidson the leptin of 3T3-L1 dipocyteswith or without wortmannin. n = 10. ^##^p < 0.01, compared with control group; **p < 0.01, compared with model group

Results showed that, in flavonoids treated IR model of 3T3-L1 adipocytes, wortmannin (2 nM) decreased the adiponectin (Fig. 8c) and leptin levels (Fig. 8d) and increased and free fatty acid level (Fig. 8b) significantly, and contributed a downward trend in the glucose consumption (Fig. 8a, p = 0.059). These results show that the inhibition of PI3K could alleviate the effect of flavonoids on the abatement of metabolic abnormalities in IR model of 3T3-L1 adipocytes.

## Discussion

The main pathogenesis of T2DM is IR, and most patients with T2DM are associated with obesity [11, 12]. Obesity increases body the volume of adipocytes and fat accumulation, which increases the release amount of FFA into the blood. Excessive FFA not only affects the storage capacity of adipocytes, but also excessively deposits in pancreas islet, skeletal muscles and liver in the form of TG [4], which injuring the function of insulin secretion from pancreatic β cells, causing IR in the liver and skeletal muscle, and leading to an increase in blood glucose in the body [4].

As a glucocorticoid, DEX can increase the intracellular FFA content, and inhibit the phosphorylation of IRS and the translocation of GLUT4 to the cell membrane surface to reduce the uptake of glucose by cells to induce the symptoms of IR [13]. In this study, we used DEX combined with insulin to induce a cell model of insulin resistance on adipocytes and explored the effects of flavonoids of mulberry leaves on it. We found that a concentration (1 μM) of DEX in combination with insulin (100 nM) resulted in a prominent symptom of IR that last at least 48 h in adipocytes. The administration of flavonoids, which extracts from mulberry leaves, could significantly increase the glucose consumption and reduce the FFA level in IR model of 3T3-L1 adipocytes. Our results confirmed that the flavonoids could alleviate the symptoms of insulin resistance in IR adipocytes.

As the most secreted cytokine in adipose tissue, studies have proved that adiponectin is a negatively correlated factor with IR and has an insulin sensitization ability [14, 15]. And leptin acts on adipocytes to inhibit the synthesis of fat and to promote the fat decomposition, which has a hypo-secretion in IR [15, 16]. Our study found that mulberry leaf extracts flavonoids could significantly increase the depressed levels of adiponect in and leptin in IR model of 3T3-L1 adipocytes.

Adipocytes plays an important role in T2DM [17], for it is the main composition of adipose tissue and the main site of IR [18]. Current study testified that disruption of the balance of insulin signaling pathway is one of the main cause of IR in adipose tissue [19, 20], and the impaired translocation ability of GLUT4 is also a cause of IR and T2DM [6, 21]. When insulin resistance existing, the body weight was increasing, and the level of FAS in adipose tissue was up-regulated [22]. Studies have shown that the inhibition of FAS might be beneficial for insulin resistance [23]. Our results showed that flavonoids that comes from mulberry leaf extracts could significantly increase the total and membrane GLUT4 in IR model of 3T3-L1 adipocytes and decreased the FAS expression, and we illuminated that this effect involves the IRS1/PI3K/AKT signaling pathway.

In the insulin conduction signaling pathway, insulin binds to the alpha and beta subunits of insulin receptor (INSR) on the membrane of adipocytes to cause auto-phosphorylation of INSR, followed by phosphorylation of multiple tyrosine residues on the IRS [24]. The phosphorylated IRS contains a SH2 domain junction, which binds to the PI3K regulatory subunit P85, activates PI3K, and regulates the downstream factor Akt, and ultimately leads to GLUT4 translocation to promote the utilization of glucose and lower body blood glucose [25]. Our results showed that mulberry leaf extracts flavonoids increased the protein expression levels of p-IRS1, p-PI3K and p-Akt, total GLUT4, and membrane GLUT4 in IR model of 3T3-L1 adipocytes. On the other hand, we found that a PI3K inhibitor, wortmannin (2 nM), partly eliminated the activation of the flavonoids on PI3K/AKT signaling and the membrane transfer capacity of GLUT4 in flavonoids treated 3T3-L1 adipocytes IR model. Furthermore, wortmannin decreased the glucose consumption and the adiponectin and leptin levels, and increased the free fatty acid level significantly in flavonoids treated 3T3-L1 adipocytes IR model.

PTEN, originally identified as a tumor suppressor, is an important regulator of the PI3K/Akt pathway [26]. The suppression of PTEN protects the mice from insulin resistance and diabetes [27]. Our results showed that mulberry leaf extracts flavonoids decreased the protein expression level of PTEN in IR model of 3T3-L1 adipocytes. The block of PI3K by wortmannin remarkably reduced the FAS expression, as well as the PI3K/AKT signaling and the membrane transfer capacity of GLUT4, but wortmannin had little influence on PTEN and p-IRS1 expressions in IR model of 3T3-L1 adipocytes when flavonoids treatment existing. The flavonoids of mulberry leaf extracts might be act as an upstream regulator of PI3K/AKT signaling. However, our experiments have not been able to determine whether it specifically targets PTEN, IRS1, or other target proteins. This is a crucial problem that needs to be addressed in our subsequent research.

In conclusion, our study illuminated that IRS1/PI3K/AKT pathway signal involved in the regulation of metabolic abnormalities by mulberry leaf extracts flavonoids in 3T3-L1 adipocytes insulin resistance model and suggested that flavonoids extracted from mulberry leaves may be a candidate for the therapeutic option for T2DM.

## Data Availability

The data used to support the findings of this study are available from the corresponding author upon request.

## Abbreviations

IRS1/PI3K/AKT: insulin receptor substrate 1/phosphoinositide 3-kinase/protein kinase B
GLUT4: glucose transporter type 4
3T3-L1: mouse embryonic fibroblast
p-IRS1: phosphorylation-Insulin receptor substrate 1
p-PI3K: phosphorylation-phosphoinositide 3-kinase
p-Akt: phosphorylation- protein kinase B
T2DM: type 2 diabetes mellitus
IR: insulin resistance
DMEM: Dulbecco’s modified Eagle’s medium
FBS: fetal bovine serum
DEX: dexamethasone
MTT: 3-[4,5-dimethylthiazol-2-yl]-2,5-diphenyltetrazolium bromide
DMSO: dimethylsulfoxide
IBMX: 3-isobutyl-1- methylxanthine
ELISA: enzyme-linked immunosorbent assay
Na–K-ATP: sodium potassium pump
PBS: phosphate-buffered saline
RIPA: radio immunoprecipitation assay
PVDF: polyvinylidene fluoride
DAPI: 4′,6-diamidino-2-phenylindole
FFA: nonesterified fatty acid
TG: triglyceride
FAS: fatty acid synthetase
PTEN: phosphatase and tensin homolog
